# Food Delivery Drivers’ Health Literacy Regarding COVID-19 Prevention and Protective Behaviors During the COVID-19 Pandemic: Cross-sectional Survey in Southern Thailand

**DOI:** 10.2196/37693

**Published:** 2022-10-13

**Authors:** Kasemsak Jandee, Chamnong Thanapop

**Affiliations:** 1 Department of Community Public Health School of Public Health Walailak University Nakhon Sri Thammarat Thailand; 2 Center of Excellence in Data Science for Health Study Walailak University Nakhon Sri Thammarat Thailand

**Keywords:** health literacy, health information, preventive behavior, COVID-19, food delivery, delivery drivers

## Abstract

**Background:**

In 2019, COVID-19 spread worldwide, causing a pandemic that has posed unprecedented challenges and pressure for health systems and economies. Food delivery services have become an important medium for consumer food purchases to limit human-to-human contact. Thus, delivery drivers are at high risk of exposure to COVID-19 infection at work. To the best of our knowledge, no studies have analyzed the dimensions of health literacy (HL) regarding COVID-19 prevention in this population.

**Objective:**

This study aims to explore the HL status toward COVID-19 prevention and its associated factors among food delivery drivers in southern Thailand.

**Methods:**

Following a cross-sectional survey from July to August 2021, Thai food delivery drivers in the upper-south and lower-south regions of southern Thailand were recruited to participate during the compulsory COVID-19 lockdown. An online structured questionnaire was administered verbally and recorded by the interviewer. Univariate and multivariate linear regressions were used to explore independently associated factors.

**Results:**

Of 401 drivers, 291 (72.6%) were men. The median age was 31 years (range 19-64 years). The median number of months working as a driver was 12 months, and the median number of working hours was 9 hours per day. The median number of daily food orders was 20, while the median daily income was Thai baht (THB) 600 (US $15.90). Social media (Facebook and Line) was a common source of health information. The most common information required was about the COVID-19 vaccine, medications, and treatment. Most drivers (285/401, 71.1%) had excellent HL levels regarding COVID-19 prevention. Only the practical application of information was statistically correlated with behavior (*r*=0.38, *P*<.001). Drivers in the lower south of Thailand were more likely to have excellent HL than other drivers (*β*=7.03, *P*<.001). Those who frequently accessed information through YouTube (*β*=–2.17, *P*=.01) and relatives (*β*=–4.19, *P*<.001) were less likely to have excellent HL levels.

**Conclusions:**

Understanding HL among food delivery drivers would be useful for planning effective interventions that target this population. Conventional health education through social media alone may not be effective at educating people about COVID-19 prevention. Information literacy skills could determine individuals’ HL and drivers’ behaviors.

## Introduction

Since the onset of the COVID-19 pandemic that has been declared a global emergency by the World Health Organization (WHO) [1,2], social distancing and self-isolation, including lockdowns, have been adopted almost universally by countries worldwide as public health and social measures to control the transmission of the virus [3]. These changes have occurred through citizens' voluntary steps and concrete government interventions. With the global recommendation of maintaining social distancing and implementing lockdowns worldwide, many businesses, especially restaurants, have been forced to close, particularly in Thailand. Thus, food delivery services have become an important medium for consumer food purchases [4,5], and the pandemic has significantly changed the food delivery service industry and consumer perceptions [6].

People generally perceive that using food delivery services is safer than going into restaurants because the limited human-to-human contact reduces the risk of SARS-CoV-2 infection [7]. A previous study revealed that consumers are less concerned about contracting COVID-19 from food in general than they are for restaurant food [8]. However, delivery drivers are a highly mobile population that offers services to a wide range of clients, including vulnerable populations, such as older adults or those less likely to leave their house for stocking up on basic needs [9]. In Thailand, there was no formal guideline or operating procedures for the drivers in the context of COVID-19 mentioned by the government or food delivery companies, and the strictness of social and public health measures was different depending on the rapid transmission of COVID-19 and cluster infections in each province.

Food delivery services in Thailand have been estimated to be worth over US $1.1 billion, with a 17% growth rate for 2020. The 4 major food delivery service providers in Thailand are GrabFood, Food Panda, Line Man, and Get [10]. Thai food delivery has been growing gradually at approximately 10% since 2017, and the COVID-19 pandemic has catalyzed growth by limiting Thai people’s ability to eat at restaurants. The pandemic has benefited Thai food delivery service operators as orders from customers have increased by 100%-300%, with at least 225,000 delivery drivers working in Thailand [11]. Drivers have suddenly been thrust into the front line of the COVID-19 pandemic. Due to the increased movement of food delivery drivers and the high number of people with whom they come into contact, these drivers are at risk of exposure to SARS-CoV-2 infection at work, similar to health care providers. Furthermore, food delivery drivers who are suspected of having contracted SARS-CoV-2 may play a role in actively transmitting the virus to consumers [12,13].

The number of COVID-19 cases among food delivery drivers has been possibly underreported by food delivery companies. A cross-sectional study conducted in Quito, Ecuador, in 2020 revealed a high incidence rate of SARS-CoV-2 infection in self-employed food delivery drivers [9]. A food delivery man in Beijing, China, was reported as having SARS-CoV-2 and had a record of delivering around 50 orders per day across a wide area in Beijing in June 2020 [14]. In addition, a previous report from a public hospital in Hanoi, Vietnam, showed that more than 60% of confirmed COVID-19 cases could be linked to food delivery at the hospital’s cafeteria [15]. Another case was reported in India, where a pizza delivery man tested positive for SARS-CoV-2. His history showed that he had contact with 72 families, including 17 other delivery men, all of whom were immediately quarantined [16]. The lack of appropriate occupational health control measures for food delivery drivers and their movements and social interactions, which are high-risk behaviors, put this population at high risk of contracting COVID-19. However, food delivery drivers are expected to play a significant role in reducing the risk of COVID-19 transmission [17].

To ensure that people adhere to infection control precautions during the COVID-19 pandemic, they must be able to access and understand public health information. People’s ability to obtain, use, and apply information to make decisions related to their health is defined as health literacy (HL) [18,19]. Many researchers have found that better public health outcomes result from people’s acquisition of new knowledge, and more positive attitudes, greater self-efficacy, and positive health behaviors are associated with higher HL [18].

Regarding HL tools, a previous systematic review showed an upward trend in the availability of tools assessing HL among the general population using multidimensional structures and comprehensive measurement approaches. However, a definite consensus could not be reached on the dimensions of HL tools [20]. Thus, an appropriate HL tool should be developed according to the specific health condition being addressed. In addition, understanding the drivers’ information needs and information-seeking behaviors is essential for developing information systems and services that adequately satisfy their needs. However, to the beset of our knowledge, there have recently been no studies exploring the dimensions of food delivery drivers’ HL toward COVID-19 prevention, even though food delivery services have been growing annually during the COVID-19 pandemic, particularly in Thailand. To investigate how food delivery drivers in southern Thailand recognize COVID-19 prevention, this study was conducted to explore their HL status toward COVID-19 prevention and its association with sociodemographic characteristics, work-related factors, and health information access. The findings from this study could provide scientific evidence for COVID-19 control and prevention programs, including suggestions for public health information campaigns for HL for food delivery services during the COVID-19 pandemic.

## Methods

### Study Design and Setting

We conducted a cross-sectional survey between July and August 2021 in southern Thailand, a long, narrow peninsula that can be further divided into an upper-south and a lower-south region [21]. Two provinces, Nakhon Sri Thammarat and Songkhla, were selected as representative of the upper-south and lower-south regions, respectively, due to the high incidence of SARS-CoV-2 infection in these provinces during the duration of this study [22]. These provinces were also selected due to the growth of food delivery services expanding from Bangkok [11]. This study was conducted using the KoBo Toolbox, a free and open source software for online and offline data collection developed by the Harvard Humanitarian Initiative [23].

### Study Participants

Thai food delivery drivers aged 18 years or older who were food delivery drivers for at least 3 months before the administration of the survey were invited to participate in the study. Those who could not read or understand Thai and those who had a communication barrier, such as being deaf or having a mental deficit, were excluded.

The required sample size was determined using the single proportion formula according to the estimated proportion of adequate HL among food delivery drivers at 50% [24] since there were no previous studies conducted in this study population in Thailand, with a 5.5% acceptable error rate and a 95% CI. Since this cross-sectional survey used convenience sampling because food delivery drivers’ interview time is a constraint, the design effect was not considered. An additional 20% of subjects were included to prevent data loss. Thus, the sample size required was at least 382 participants. The study met this requirement and included 401 participants (401/417, 96.2% response rate). Through intensive outreach with the snowball sampling technique in each region, the recruitment process was initiated by spreading information about the study through a group of food delivery drivers. The first interested individual was invited to participate in this study and helped us identify further potential participants. This step was repeated until the needed sample size was found.

### Instrument and Measurements

A structured questionnaire was developed to assess the HL status of Thai food delivery drivers regarding COVID-19 prevention. The questionnaire consisted of sets of questions to determine participants’ demographics, work-related factors, health information access, understanding, judgment, and application of health information toward COVID-19 prevention, including preventive behaviors. The questionnaire was reviewed by 3 public health experts, who rated the overall content validity of the questionnaire at 1.00, where the index of item objective congruence was over 0.5. A pilot test of the survey instruments was then conducted among 30 participants prior to administering the survey. The pilot study returned acceptable reliability values for each dimension of HL (0.73-0.81). The pilot study survey was verbally administered in the Nakhon Sri Thammarat province in southern Thailand, and the interviewer recorded responses on electronic handheld devices using KoBo technology [23]. This device can be used for data collection offline and then synchronized onto a central database when telephone signals or wireless networks are available. It should be noted that the personal information was treated confidentially within the system applications during the study period.

Questions regarding demographic characteristics included age, sex, education, food delivery company, religion, income, years/months of experience in food delivery services, number of hours worked per day, and number of completed food orders delivered per day. A single letter was used to represent each food delivery company's name to avoid disclosure of the official name due to this being a sensitive issue resulting in a competitive advantage. Questions on current health status included pre-existing conditions, cigarette smoking, and alcohol consumption. Participants’ sources of health information were requested to determine their recent information access about COVID-19 prevention. The 12 questions regarding understanding were yes/no questions, where correct answers were given a score of 1, while incorrect answers were given a score of 0. Therefore, the participants’ understanding scores ranged from 0 to 12. A set of questions to measure how the participants make decisions when confronted with COVID-19 prevention information was used to indicate the degree of participants’ ability to judge/make decisions on each statement. Participants were asked to rate 6 questions on a 5-point Likert scale, with the responses being “extremely easy” (5), “slightly easy” (4), “neutral” (3), “slightly difficult” (2), and “extremely difficult” (1). Participants’ ability to apply health information to their profession was assessed using 5 questions. Items were answered using a 5-point Likert scale from 1 (never) to 5 (always); total scores ranged from 5 to 25. The frequency of certain COVID-19 prevention behaviors was assessed using a 5-point Likert scale: “never” (1), “seldom” (2), “sometimes” (3), “often” (4), and “always” (5). COVID-19 prevention behavior scores ranged from 6 to 30. The total score for each section was summarized, and HL levels were classified according to the criteria. As there is no specific guideline for how to classify HL levels [20,25], the overall HL levels and other dimensions, including the levels of preventive behaviors, were classified as excellent (score≥80%), moderate (score=60%-79%), and inadequate (score<60%).

### Statistical Analysis

Demographic, work-related, and health information–seeking factors of food delivery drivers were descriptively presented as percentages, mean (SD), or median (IQR). HL was determined using the sum of the scores from the understanding, judgment/decision-making, and application of information questions. Access to COVID-19 prevention information was described in terms of health information sources commonly used by the participants. This information was not included in the overall HL score.

Associations between the HL score, preventive behavior score, and independent variables were assessed using univariate/multivariate linear regression. A stepwise multiple linear regression model was used to determine the most significant predictors of HL and behavior toward COVID-19 prevention. We described the strength of the measure of association using the mean difference in the regression analysis. Correlation analyses between the components of HL and preventive behaviors among drivers were performed using the Pearson correlation coefficient (r). Differences were considered statistically significant at a *P* value of .05. All statistical analyses were performed using R version 4.1.2 statistical analysis software (R Foundation for Statistical Computing).

### Ethical Considerations

This study was reviewed and approved by the Human Research Ethics Committee of Walailak University, Thailand, and followed the principles of the Declaration of Helsinki (WUEC-21-071-01). All participants were informed of all details regarding the study, and informed consent was obtained before the participants completed the online form.

## Results

### Demographic and Work-Related Factors

A total of 401 participants completed an online questionnaire survey, and nearly three-quarters of them (291/401, 72.6%) were male. Demographic characteristics and work-related factors of the participants are presented in Table 1. The participants’ age ranged from 19 to 64 years, with a median age of 31 years. Of the 401 participants, 344 (85.8%) were Buddhist, and most of the participants’ education level was high school or lower. Company A represents the highest category of food delivery company. Participants reported that they were food delivery drivers for a median of 12 months. The median number of working hours was 9 hours per day, the median number of food order deliveries per day was 20, and participants’ median daily income was Thai baht (THB) 600 (US $15.90). Most participants’ (241/401, 60.1%) health insurance status was a universal health coverage scheme. More than half of the participants did not smoke (245/401, 61.1%) or consume alcohol (272/401, 67.8%). In addition, 21 (5.2%) of the participants had pre-existing chronic illnesses. The distributions of these characteristics among participants in the upper-south (n=201, 50.1%) and lower-south (n=200, 49.9%) regions of Thailand were almost all different, excluding pre-existing chronic illness status and alcohol consumption, as shown in Table 1. Regarding work-related factors, participants in the lower south of Thailand were more likely to have a longer working duration than those in the upper-south region (12 vs 8 months, *P*<.001). In contrast, participants in the upper south of Thailand were more likely to deliver a higher number of food orders (20 vs 18 deliveries, *P*<.001) and earn daily income (THB 600 vs 500 [US $15.90 vs $13.25], *P*<.001) than those in lower-southern Thailand.

**Table 1:** Demographic characteristics and work-related factors of participants.

Characteristics		Total (N=401)	Upper south (n=201)	Lower south (n=200)
**Sex, n (%), *P*^a^=.002**				
	Male	291 (72.6)	160 (79.6)	131 (65.5)
	Female	110 (27.4)	41 (20.4)	69 (34.5)
**Age (years), *P*<.001**				
	Median (IQR)	31 (24-40)	27 (23-34)	36 (28-46)
**Religion, n (%), *P*<.001**				
	Buddhism	344 (85.8)	186 (92.5)	158 (79.0)
	Islam	54 (13.5)	13 (6.5)	41 (20.5)
	Christian	3 (0.7)	2 (1.0)	1 (0.5)
**Education, n (%), *P*=.004**				
	High school or below	288 (71.8)	131 (65.2)	157 (78.5)
	Bachelor or above	113 (28.2)	70 (34.8)	43 (21.5)
**Food delivery company, n (%), *P*<.001**				
	A	188 (46.9)	102 (50.7)	86 (43.0)
	B	148 (36.9)	38 (18.9)	110 (55.0)
	C	60 (15.0)	56 (27.9)	4 (2.0)
	D	5 (1.2)	5 (2.5)	0
**Working duration as driver (months), *P*<.001**				
	Median (IQR)	12 (5-17)	8 (4-15)	12 (7-24)
**Working hour per day, *P*=.004**				
	Median (IQR)	9 (8-10)	8 (7-10)	9 (8-12)
**Number of daily food order deliveries, *P*<.001**				
	Median (IQR)	20 (15-25)	20 (20-30)	18 (15-20)
**Daily income (THB^b,c^), *P*<.001**				
	Median (IQR)	600 (500-700), or US $15.90 ($13.25-$18.55)	600 (500-700), or US $15.90 ($13.25-$18.55)	500 (400-600), or US $13.25 ($10.60-$15.90)
**Health insurance, n (%), *P*=.04**				
	Universal coverage	241 (60.1)	120 (59.7)	121 (60.5)
	Civil servant	4 (1.0)	4 (2.0)	0
	Social security	113 (28.2)	50 (24.9)	63 (31.5)
	Self-pay	43 (10.7)	27 (13.4)	16 (8.0)
**Pre-existing chronic illnesses, n (%), *P*=.66**				
	No	380 (94.8)	189 (94.0)	191 (95.5)
	Yes	21 (5.2)	12 (6.0)	9 (4.5)
**Cigarette smoker, n (%), *P*<.001**				
	Never smoke	245 (61.1)	124 (61.7)	121 (60.5)
	Former smoker	59 (14.7)	42 (20.9)	17 (8.5)
	Current smoker	97 (24.2)	35 (17.4)	62 (31.0)
**Alcohol consumption, n (%), *P*=.99**				
	Never drink	272 (67.8)	136 (67.7)	136 (68.0)
	Current drinker	129 (32.2)	65 (32.3)	64 (32.0)
**COVID-19 HL^d^ levels, n (%), *P*<.001**				
	Excellent	285 (71.1)	91 (45.3)	194 (97.0)
	Moderate	98 (24.4)	92 (45.8)	6 (3.0)
	Inadequate	18 (4.5)	18 (8.9)	0
**COVID-19 preventive behavior levels, n (%), *P*<.001**				
	Excellent	355 (88.5)	165 (82.1)	190 (95.0)
	Moderate	45 (11.2)	35 (17.4)	10 (5.0)
	Inadequate	1 (0.3)	1 (0.5)	0

^a^*P* value: chi-square test for categorical outcomes and Mann-Whitney test for numerical outcomes.

^b^THB: Thai baht.

^c^An exchange rate of THB 1=US $0.026 has been applied.

^d^HL: health literacy.

### Health Information Needs Regarding COVID-19

**Figure 1:**
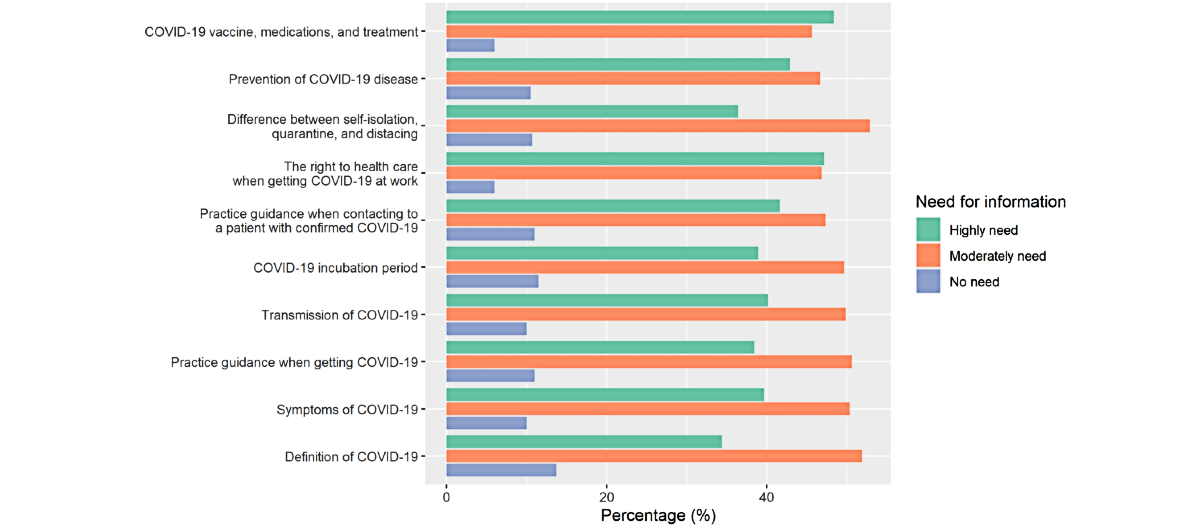
Participants’ COVID-19 information needs.

The participants’ health information needs regarding COVID-19 are shown in Figure 1. The participants’ most common health information needs regarding COVID-19 were related to vaccines, medications, and treatment (194/401, 48.4%), followed by the right to health care when getting COVID-19 at work (189/401, 47.1%). The least needed information was the definition of COVID-19 (55/401, 13.7%) and the COVID-19 incubation period (46/401, 11.5%).

### Health Literacy Toward COVID-19

Four main dimensions of HL regarding COVID-19 were identified: access to information, understanding, decision/judgment, and applying information, which included COVID-19 prevention behaviors. Regarding accessibility to health information related to COVID-19, the social media site Facebook was the most common source of information, followed by Line; 222 (55.4%) and 206 (51.4%) of 401 participants reported that they sometimes accessed information via websites and YouTube, respectively (Table 2), while 147 (36.7%) of the participants often accessed information from broadcasts, such as television. In addition, three-fourths of the participants revealed that they had heard of COVID-19 from relatives and colleagues. The information resources accessed by participants differed between the 2 regions of southern Thailand. The lower-south region had a significantly higher frequency of access to all resources, as presented in Table 2.

Of the 401 participants, 380 (94.8%) perceived that they had sufficient knowledge about COVID-19 (Table 3). A majority (393/401, 98%) obtained information and notifications regarding COVID-19 from their food delivery companies. Most participants (393/401, 98%) reported that they could easily access information about handwashing and mask wearing, although 326 (81.3%) participants were able to access that information less frequently than weekly. In addition, 380 (94.8%) participants usually verified the handwashing and mask-wearing information.

**Table 2:** Information resources regarding COVID-19 prevention.

Information resources and frequency of access		Total (N=401), n (%)	Upper south (n=201), n (%)	Lower south (n=200), n (%)
**Website, *P*^a^<.001**				
	Frequent	45 (11.2)	36 (17.9)	9 (4.5)
	Sometimes	222 (55.4)	97 (48.3)	125 (62.5)
	Never	134 (33.4)	68 (33.8)	66 (33.0)
**Line, *P*<.001**				
	Frequent	196 (48.9)	127 (63.2)	69 (34.5)
	Sometimes	182 (45.4)	59 (29.3)	123 (61.5)
	Never	23 (5.7)	15 (7.5)	8 (4.0)
**Facebook, *P*=.01**				
	Frequent	299 (74.6)	140 (69.6)	159 (79.5)
	Sometimes	86 (21.4)	48 (23.9)	38 (19.0)
	Never	16 (4.0)	13 (6.5)	3 (1.5)
**YouTube, *P*<.001**				
	Frequent	101 (25.2)	68 (33.8)	33 (16.5)
	Sometimes	206 (51.4)	54 (26.9)	152 (76.0)
	Never	94 (23.4)	79 (39.3)	15 (7.5)
**Television, *P*<.001**				
	Frequent	147 (36.7)	17 (8.5)	130 (65.0)
	Sometimes	120 (29.9)	59 (29.3)	61 (30.5)
	Never	134 (33.4)	125 (62.2)	9 (4.5)
**Relatives, *P*<.001**				
	Frequent	146 (36.4)	16 (8.0)	130 (65.0)
	Sometimes	149 (37.2)	80 (39.8)	69 (34.5)
	Never	106 (26.4)	105 (52.2)	1 (0.5)
**Colleagues, *P*<.001**				
	Frequent	159 (39.7)	22 (10.9)	137 (68.5)
	Sometimes	150 (37.4)	87 (43.3)	63 (31.5)
	Never	92 (22.9)	92 (45.8)	0

^a^*P* value: chi-square test.

The understanding, judgment, and application of information regarding COVID-19 prevention were assessed using a set of questions. Participants’ understanding of COVID-19 prevention is shown in Table 4. More than 90% (360/401) of the participants correctly answered 10 (83%) of the 12 questions. Interestingly, 157 (39.2%) drivers did not know that they should change their masks daily. Although the participants’ ability to judge information and make decisions regarding COVID-19 prevention varied, most (362/401, 90.3%) participants reported that they were able to exchange information about COVID-19 prevention measures with health care providers and their colleagues, and 360 (89.8%) participants had decided to wear a mask and wash their hands every time they delivered food to a customer. In contrast, 132 (32.9%) drivers found it extremely difficult to question health care providers when they were confused about proper mask wearing and handwashing, as shown in Table 5.

Regarding the application of information, the majority (373/401, 93%) of the participants reported that they always keep a distance of at least 1 m from others when standing or sitting at a restaurant and when delivering food to customers. Almost all (348/401, 86.8%) participants monitored themselves and their families for COVID-19–related symptoms (Table 6). However, 69 (17.2%) participants did not use, or seldom used, a Food and Drug Administration (FDA)–certified mask. COVID-19 prevention behaviors are presented in Table 6. Most (395/401, 98.5%) participants reported that they always clean their hands using either regular soap and water or an alcohol-based hand rub before and after delivering food to consumers, and they wear a mask regularly while in public places, particularly when picking up and delivering food/beverage orders (390/401, 97.3%). Interestingly, 269 (67.1%) participants reported that instead of taking their masks off, they always pull them down under the chin to talk and to eat or drink.

**Figure 2:**
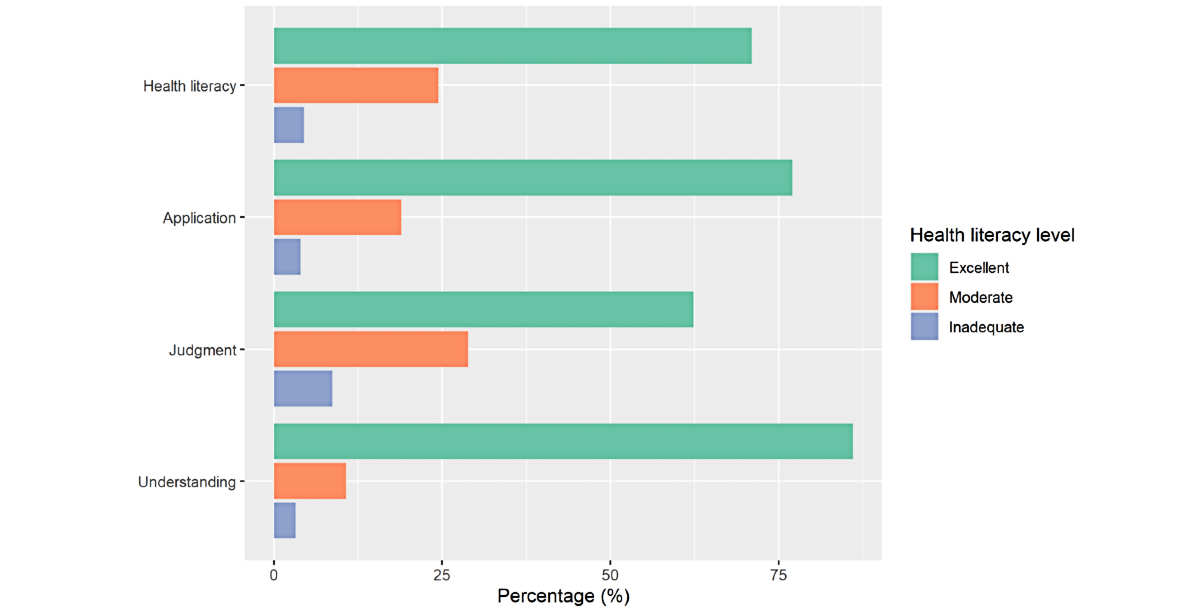
Participants’ health literacy levels on each dimension.

Drivers’ distributions according to the 3 dimensions (understanding, judgment, and application) are shown in Figure 2. Most participants had excellent HL levels in all 3 dimensions. Of the 401 participants, 345 (86%) had an excellent understanding of COVID-19, while 13 (3.2%) had an inadequate understanding. Excellent and moderate judgment accounted for 62.3% (250/401) and 28.9% (116/401) of the participants, respectively. Of the 401 participants, 309 (77.1%) were rated “excellent” at applying COVID-19 information to preventive behaviors, whereas 16 (4%) were rated “inadequate.” A large proportion (285/401, 71.1%) of drivers had excellent HL, followed by moderate (98/401, 24.4%) and inadequate (18/401, 4.5%) HL. Most (355/401, 88.5%) of the participants had excellent COVID-19 prevention behavior. The difference in the level of each dimension of HL was statistically significant between participants in 2 different southern regions of Thailand. Participants in the lower south were more likely to have excellent HL and preventive behavior levels than those in the upper south (Table 1).

**Table 3:** Factors related accessibility to health information regarding COVID-19 prevention.

Variables			Total (N=401), n (%)	Upper south (n=201), n (%)	Lower south (n=200), n (%)
**Perceived sufficient knowledge regarding COVID-19 prevention, *P*<.001**					
	Yes	380 (94.8)		181 (90.0)	199 (99.5)
	No	21 (5.2)		20 (10.0)	1 (0.5)
**Obtaining information regarding COVID-19 prevention from the company, *P*=.28**					
	Yes	393 (98.0)		195 (97.0)	198 (99.0)
	No	8 (2.0)		6 (3.0)	2 (1.0)
**Ability to access information regarding handwashing and mask wearing, *P*=.07**					
	Access	393 (98.0)		194 (96.5)	199 (99.5)
	No access	8 (2.0)		7 (3.5)	1 (0.5)
**Frequency of seeking information, *P*<.001**					
	Less than weekly	326 (82.1)		135 (68.2)	191 (96.0)
	Weekly	71 (17.9)		63 (31.8)	8 (4.0)
**Information verification, *P*=.13**					
	Yes	380 (95.7)		186 (93.9)	194 (97.5)
	No	17 (4.3)		12 (6.1)	5 (2.5)

**Table 4:** Understanding of drivers (N=401) regarding COVID-19 prevention.

Variables	Yes, n (%)	No, n (%)
COVID-19 is an infectious disease caused by the SARS-CoV-2, which has been found since 2019.	380 (94.8)	21 (5.2)
The most common symptoms of COVID-19 include cough and tiredness.	385 (96.0)	16 (4.0)
Older adults, including those with diabetes/hypertension/heart diseases/chronic lung disease/cancers, are at the highest risk of COVID-19–related adverse outcomes and mortality.	382 (95.3)	19 (4.7)
COVID-19 patients with or without symptoms can pass on the virus.	384 (95.8)	17 (4.2)
If people feel sick or have trouble breathing, they should go to see a doctor immediately.	375 (93.5)	26 (6.5)
The spread of COVID-19 occurs via airborne particles and droplets when they exhale (eg, speaking, coughing, and sneezing).	382 (95.3)	19 (4.7)
COVID-19 infection is possible by touching contaminated surfaces (eg, doorknobs, handles, and tables).	382 (95.3)	19 (4.7)
People should be self-quarantined for 14 days after their last contact with infected patients and monitor themselves for fever, cough, etc.	390 (97.3)	11 (2.7)
Mask wearing can prevent the transmission of COVID-19.	361 (90.0)	40 (10.0)
Changing masks daily is effectively preventive behavior.	244 (60.8)	157 (39.2)
A cloth face mask should be washed at least once a day.	350 (87.3)	51 (12.7)
Handwashing following 7 steps and for at least 20 seconds is an effective process.	377 (94.0)	24 (6.0)

**Table 5:** Judgment of information toward COVID-19 prevention among drivers (N=401).

Variables	Extremely easy/slightly easy	Neutral	Extremely difficult/slightly difficult
Question health care providers when you are confused about proper mask wearing and handwashing.	243 (60.6)	26 (6.5)	132 (32.9)
Question about the effective ways of preventing novel coronavirus infection from health care providers and others.	251 (62.6)	22 (5.5)	128 (31.9)
Decide how to correctly wear a mask and wash hands when delivering food to consumer.	342 (85.3)	27 (6.7)	32 (8.0)
Decide to wear a mask and wash hands every time when delivering food to each consumer.	360 (89.8)	27 (6.7)	14 (3.5)
Know the advantages and disadvantages of each preventive measure when working as a food delivery driver.	331 (82.5)	45 (11.2)	25 (6.2)
Exchange information about COVID-19 prevention measures with health care providers, including colleagues at work.	362 (90.3)	28 (7.0)	11 (2.7)

**Table 6:** Application of information regarding COVID-19 prevention and preventive behaviors among drivers (N=401).

Variables			Often/always		Sometimes		Never/seldom
**Application of information toward COVID-19 prevention**							
	Monitor yourself and your family for COVID-19–related symptoms (eg, fever, cough, trouble breathing) and suggest seeing a doctor immediately when getting these symptoms.	348 (86.8)		31 (7.7)		22 (5.5)	
	Consider using an appropriate mask by yourself when working as a food delivery driver.	341 (85.0)		31 (7.7)		29 (7.2)	
	Use a mask certified by the Food and Drug Administration (FDA).	292 (72.8)		40 (10.0)		69 (17.2)	
	Wash your hands following the 7 steps of effective handwashing.	345 (86.0)		34 (8.5)		22 (5.5)	
	Keep a distance of at least 1 m from others when standing or sitting at a restaurant and delivering food to consumers.	373 (93.0)		19 (4.7)		9 (2.3)	
**Preventive behaviors toward COVID-19**							
	Replace a mask with a new one once it is wet or soiled from saliva or mucus.	384 (95.8)		10 (2.5)		7 (1.7)	
	Pull the mask down under the chin to talk to and to eat or drink instead of taking the mask off.	269 (67.1)		35 (8.7)		97 (24.2)	
	Wash your hands before touching your face, eyes, nose, or mouth.	349 (87.0)		42 (10.5)		10 (2.5)	
	Clean your hands immediately with soap and water or an alcohol-based hand rub after touching doorknobs, handrails, light switches, and much more.	379 (94.5)		19 (4.7)		3 (0.8)	
	Wear a mask regularly when in public places (eg, market, restaurant), particularly when picking up and delivering food/beverage orders.	390 (97.3)		9 (2.2)		2 (0.5)	
	Clean your hands regularly with soap and water or an alcohol-based hand rub before and after delivering food to consumers.	395 (98.5)		4 (1.0)		2 (0.5)	

**Figure 3:**
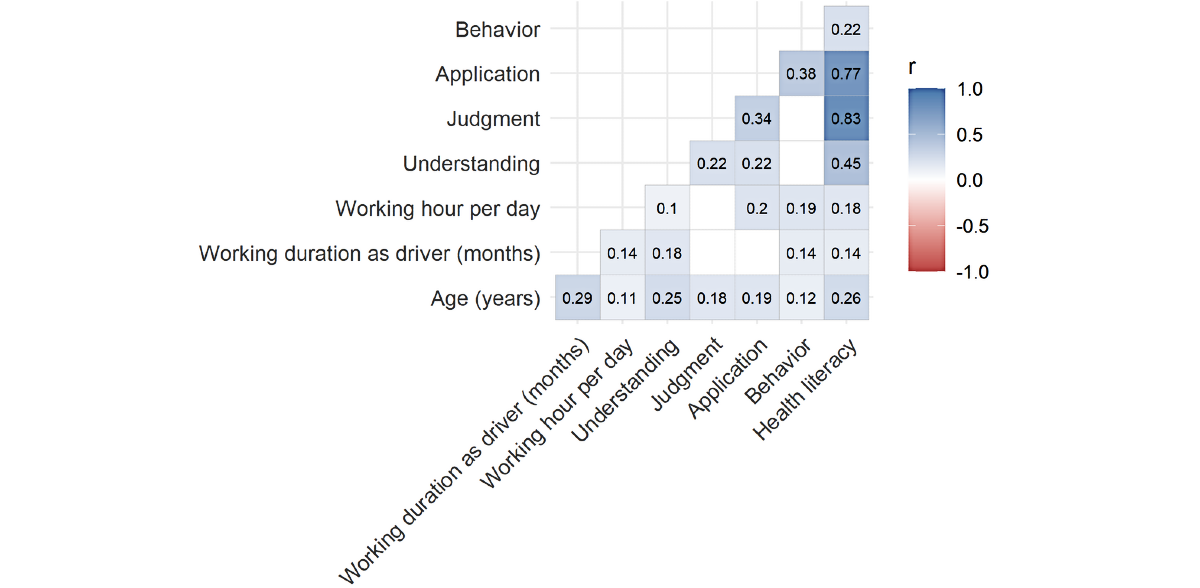
Correlation matrix of research variables.

The Pearson moment correlation coefficient (r) was used to examine the relationship between participants’ age, work duration (months), number of working hours per day, understanding, judgment, application, and behavior scores. Figure 3 shows weak-to-strong correlation coefficients for the predictor variables associated with the HL of participants. The relationships between judgment and HL and between application and HL were positively correlated and statistically significant (*r*=0.83 and 0.77, respectively); a statistically significant moderate positive correlation was found between understanding and HL (*r*=0.45). These results suggest that higher scores on participants’ judgment and application of information are associated with higher HL scores. Furthermore, a significant moderate positive correlation was found between participants’ application of information and behavior toward COVID-19 prevention (*r*=0.38), whereas a weak positive correlation was found between HL and behavior (*r*=0.22). Meanwhile, age was significantly weakly positively correlated with work duration (*r*=0.29), understanding (*r*=0.25), and HL (*r*=0.26).

Associations between factors, HL, and behavior toward COVID-19 prevention were determined using univariate and multivariate analyses and are presented in Table 7. HL and behavior scores were treated as continuous variables; subsequently, they were analyzed using 2 models, HL and behavior models. All significant factors from the univariate analysis were included in the multivariate analysis in the HL model. Results from the multivariate analysis showed factors significantly associated with HL regarding COVID-19, which were the region participants worked in, health insurance status (social security scheme), information resource access (YouTube, television, and relatives), knowledge of COVID-19, and frequency of seeking information. Participants in the lower-south region of Thailand had higher HL than those in the upper-south region (*P*<.001). Participants who frequently accessed information via television, and had knowledge of COVID-19, had significantly higher HL than the others. In contrast, participants who belonged to self-payment health care, those who accessed information through YouTube and relatives, and those who frequently accessed information less than weekly had significantly lower HL than the others.

For a multivariable model of COVID-19 prevention behavior, the region participants worked in, their sex, number of working hours per day, information resources (Facebook), information verification, and HL score were significantly associated with their COVID-19 prevention behavior. The COVID-19 prevention behavior of men was statistically significantly lower than that of women. Participants who accessed information through Facebook had higher COVID-19 prevention behavior than those who never accessed it; those who verified information before its application also had higher behavior than those who did not verify information. Additionally, higher HL was significantly associated with higher COVID-19 prevention behaviors.

**Table 7:** Univariate and multivariate regression analyses demonstrating associations between independent variables and HL score and behavior score regarding COVID-19 prevention.

Characteristic			HL^a^ score univariate analysis				HL score multivariate analysis				Behavior score univariate analysis				Behavior score multivariate analysis		
			Crude coefficient (95% CI)		*P* value		Adjusted coefficient^b^ (95% CI)		*P* value		Crude coefficient (95% CI)		*P* value		Adjusted coefficient^b^ (95% CI)		*P* value
Region (lower south vs upper south)			6.44 (5.26-7.62)		<.001		7.03 (5.36-8.7)		<.001		1.36 (0.79-1.92)		<.001		0.81 (0.17-1.45)		.01
Sex (female vs male)			0.99 (–0.51 to 2.49)		.20		N/A^c^		N/A		–0.42 (–1.07 to 0.23)		.20		–0.76 (–1.38 to –0.13)		.02
Age (years)			0.18 (0.11-0.24)		<.001		N/A		N/A		0.03 (0.01-0.06)		.01		N/A		N/A
**Religion (reference=Buddhism)**																	
	Islam	0.95 (–1.02 to 2.91)		.34		N/A		N/A		0.35 (–0.5 to 1.21)		.40		N/A		N/A	
	Christian	5.75 (–1.98 to 13.47)		.14		N/A		N/A		–0.31 (–3.67 to 3.05)		.86		N/A		N/A	
Education (bachelor or above vs high school or below)			–2.00 (–3.48 to –0.53)		.01		N/A		N/A		0.15 (–0.5 to 0.79)		.66		N/A		N/A
**Food delivery company (reference=company A)**																	
	B	1.64 (0.18-3.1)		.03		N/A		N/A		1.03 (0.4-1.66)		.001		N/A		N/A	
	C	–0.87 (–2.86 to 1.13)		.39		N/A		N/A		–0.01 (–0.87 to 0.85)		.99		N/A		N/A	
	D	–1.42 (–7.43 to 4.58)		.64		N/A		N/A		–0.92 (–3.51 to 1.67)		.49		N/A		N/A	
Working duration as driver (months)			0.11 (0.03-0.19)		.01		N/A		N/A		0.05 (0.01-0.08)		.01		N/A		N/A
Working hour per day			0.46 (0.2-0.71)		<.001		N/A		N/A		0.22 (0.11-0.33)		<.001		0.17 (0.06-0.28)		.002
Number of daily food order deliveries			–0.11 (–0.21 to –0.02)		.02		N/A		N/A		–0.02 (–0.07 to 0.02)		.24		N/A		N/A
Daily income (THB^d^)			0 (–0.01 to 0)		.16		N/A		N/A		0 (0-0)		.99		N/A		N/A
Pre-existing chronic illnesses (yes vs no)			–0.35 (–3.34 to 2.65)		.82		N/A		N/A		–0.78 (–2.08 to 0.52)		.24		N/A		N/A
**Cigarette smoker (reference=never smoke)**																	
	Former smoker	–1.33 (–3.26 to 0.61)		.18		N/A		N/A		–0.23 (–1.08 to 0.61)		.59		N/A		N/A	
	Current smoker	1.77 (0.17-3.37)		.03		N/A		N/A		0.40 (–0.29 to 1.1)		.26		N/A		N/A	
Alcohol consumption (current drinker vs never drink)			0.39 (–1.05 to 1.82)		.60		N/A		N/A		0.15 (–0.47 to 0.78)		.63		N/A		N/A
**Health insurance status (reference=universal coverage)**																	
	Civil servant	–6.2 (–12.75 to 0.35)		.06		–1.45 (–6.63 to 3.73)		.58		–0.54 (–3.47 to 2.38)		.71		N/A		N/A	
	Social security	–0.04 (–1.53 to 1.45)		.96		0.49 (–0.69 to 1.67)		.41		–0.12 (–0.79 to 0.54)		.71		N/A		N/A	
	Self-pay	–4.96 (–7.11 to –2.8)		<.001		–2.25 (–3.98 to –0.53)		.01		–0.58 (–1.54 to 0.38)		.23		N/A		N/A	
**Website (reference=never)**																	
	Frequent	0.03 (–2.3 to 2.36)		.98		N/A		N/A		–0.46 (–1.47 to 0.54)		.37		N/A		N/A	
	Sometimes	0.05 (–1.42 to 1.52)		.95		N/A		N/A		0.46 (–0.17 to 1.09)		.15		N/A		N/A	
**Line (reference=never)**																	
	Frequent	1.31 (–1.68 to 4.29)		.39		N/A		N/A		–0.01 (–1.31 to 1.29)		.99		N/A		N/A	
	Sometimes	2.69 (–0.3 to 5.69)		.08		N/A		N/A		0.43 (–0.88 to 1.73)		.52		N/A		N/A	
**Facebook (reference=never)**																	
	Frequent	0.17 (–3.25 to 3.58)		.92		N/A		N/A		1.92 (0.44-3.39)		.01		1.66 (0.24-3.08)		.02	
	Sometimes	–1.35 (–4.98 to 2.29)		.47		N/A		N/A		1.50 (–0.06 to 3.07)		.06		1.35 (–0.15 to 2.86)		.08	
**YouTube (reference=never)**																	
	Frequent	–3.09 (–4.99 to –1.2)		.001		–2.17 (–3.88 to –0.47)		.01		0.38 (–0.45 to 1.21)		.37		N/A		N/A	
	Sometimes	–0.53 (–2.19 to 1.12)		.53		–4.08 (–5.72 to –2.45)		<.001		0.63 (–0.1 to 1.35)		.09		N/A		N/A	
**Television (reference=never)**																	
	Frequent	5.58 (4.08-7.08)		<.001		2.81 (0.95-4.66)		.003		1.29 (0.61-1.97)		<.001		N/A		N/A	
	Sometimes	1.18 (–0.4 to 2.75)		.14		1.54 (–0.11 to 3.19)		.07		0.40 (–0.32 to 1.13)		.27		N/A		N/A	
**Relatives (reference=never)**																	
	Frequent	3.03 (1.42 to 4.64)		<.001		–4.19 (–6.25 to –2.13)		<.001		0.86 (0.12-1.6)		.02		N/A		N/A	
	Sometimes	–2.54 (–4.14 to –0.93)		.002		–5.36 (–7.1 to –3.63)		<.001		–0.07 (–0.8 to 0.67)		.86		N/A		N/A	
**Colleagues (reference=never)**																	
	Frequent	2.72 (1.02-4.42)		.002		N/A		N/A		1.22 (0.47-1.97)		.002		N/A		N/A	
	Sometimes	–1.43 (–3.14 to 0.29)		.10		N/A		N/A		0.15 (–0.61 to 0.91)		.69		N/A		N/A	
Perceived sufficient knowledge of COVID-19 (yes vs no)			5.35 (2.25-8.44)		<.001		4.61 (2.1-7.12)		<.001		0.72 (–0.63 to 2.08)		.30		N/A		N/A
Obtaining information from the company (yes vs no)			3.33 (–2.67 to 9.33)		.28		N/A		N/A		0.90 (–1.7 to 3.51)		.50		N/A		N/A
Accessibility to information (access vs no access)			5.83 (1.1-10.57)		.02		2.89 (–0.81 to 6.59)		.13		1.21 (–0.85 to 3.28)		.25		N/A		N/A
Frequency of seeking information (weekly vs less than weekly)			–6.59 (–8.22 to –4.97)		<.001		–3.04 (–4.56 to –1.52)		<.001		–1.13 (–1.88 to –0.38)		.003		N/A		N/A
Information verification (yes vs no)			1.82 (–1.49 to 5.12)		.28		N/A		N/A		2.01 (0.59-3.43)		.01		1.42 (0.05-2.79)		.04
HL score			N/A		N/A		N/A		N/A		0.10 (0.05-0.14)		<.001		0.06 (0.01-0.1)		.02

^a^HL: health literacy.

^b^Adjusted by the backward stepwise method.

^c^N/A: not applicable.

^d^THB: Thai baht.

## Discussion

### Principal Findings

Food delivery drivers are at high risk of exposure to SARS-CoV-2 infection at work, and those who are suspected of contracting the infection might play a role in actively transmitting the infection to customers [12,15]. This study is the first cross-sectional survey on comprehensive HL related to COVID-19 prevention among food delivery drivers in southern Thailand. We aimed to explore drivers’ information needs and resources and assess their HL levels regarding COVID-19 prevention.

The findings of this study revealed that almost half of food delivery drivers need to know about vaccines, medications, treatment for COVID-19, as well as the right to health care when getting SARS-CoV-2 infection. Participants were less concerned about the natural history of COVID-19 and its definition. Various studies conducted among medical learners and undergraduate and graduate students in Jordan [26,27], senior pharmacy students at the British University in Egypt [28], and internet users and residents in China [29,30] have revealed that participants use social media platforms as their primary source of information about COVID-19. These results were consistent with our findings that social media, such as Facebook and Line, was the most common source of information regarding COVID-19 prevention used by drivers. These sources of information tend to be common for every aspect of daily life during working hours. However, health information from social media platforms may only provide broad and nonspecific information about COVID-19 prevention. Drivers may need specific and customized information related to their lifestyle, especially information regarding the right to health care when getting a SARS-CoV-2 infection, which they can get from local health care workers and their companies through social media platforms with user-friendly content, for instance, eye-catching images that separate text. More importantly, Thai government authorities and food delivery companies should work more on clarifying the rights and responsibilities of drivers when they are at high risk of COVID-19 infection during working hours by improving relevant policies and regulations. Building effective daily routine online communication for drivers is key to successfully increasing their HL levels and preventive behaviors. A closed-class Facebook group can be used as an alternative learning support application regarding COVID-19 prevention because it easily retrieves information sources and shares them with colleagues for intellectual discussion [31].

Our study also found that relatives and colleagues played a role in disseminating information to the drivers. However, health workers have been reported to be a source that can provide accurate information in an institutional-based cross-sectional study among university students in Colombia [32]. The majority of drivers in our study showed that they usually obtained and verified information through any resource, particularly social media. A previous review by González-Padilla and Tortolero-Blanco [33] presented the advantages and disadvantages associated with the use of social media platforms during the pandemic. Important disadvantages are that invalid or outdated information is common, and information on social media platforms is often not fact-checked. People should be aware of these disadvantages when seeking information via social media. An important advantage is the rapid dissemination of educational content. Thus, effective communication via accessible information resources, such as social media platforms, including the frequency of health information delivery, may help improve drivers’ understanding of COVID-19 prevention.

The 4 dimensions of HL are competencies in health information processing, including accessing, understanding, decision-making/judging, and applying information. These overall competencies subsequently influence individual health behaviors [19]. HL is an important factor in improving the understanding, risk awareness, and decision-making regarding preventive behaviors and lifestyles during a pandemic [34,35]. We did not find any studies that have reported the status of HL regarding COVID-19 prevention among food delivery drivers. Most drivers in this study had excellent HL regarding COVID-19 prevention, with only a few having inadequate HL. Various researchers have indicated that better health outcomes are associated with higher HL, which results from the acquisition of new knowledge; higher HL is also associated with more positive attitudes, greater self-efficacy, and positive health behaviors [18]. We found that it was possible to develop the HL of drivers from the beginning, and the drivers accessed information regarding COVID-19 prevention often, mostly via social media. The participants had excellent levels of understanding, judgment, and application of information regarding COVID-19 prevention, which translates into excellent HL. In the multivariate analysis, drivers in the lower south of Thailand were more likely to have excellent HL levels than those in the upper south. Food delivery services were established in the lower-south region of Thailand in 2019 and started in the upper-south region a year later. During the study period, the Thai government declared a national state of emergency in an intensified attempt to stun the spread of the coronavirus. Thailand’s Center for COVID-19 Situation Administration (CCSA) categorizes provinces into dark-red, red, and orange zones in decreasing order of the strictness of social and public health measures. The lower-south region of Thailand was categorized as dark red because of the rapid transmission of COVID-19 and cluster infections. This region exercised social distancing, lockdowns, and a nighttime curfew, as prescribed through concrete government interventions. Food delivery drivers needed to work according to strict regulations during the pandemic to mitigate their risk of SARS-CoV-2 infection [36]. Public health promotion campaigns have also been established in the lower south of Thailand, including a COVID-19 vaccination campaign. The regulations and campaigns in the lower-south region may have led to a higher HL level among drivers in the lower south than among those who work in the upper south of Thailand.

We found that the frequency of seeking information and information resources, such as YouTube and relatives, is negatively associated with HL among drivers. These findings were in contrast to those from a survey of Japanese people that showed that higher literacy is positively associated with health information access and obtaining sufficient information from multiple sources [37]. These findings were also in contrast to a cross-sectional study among Thai older adults that showed that the more access older adults have to health information, the higher is their HL [38]. However, not only informational accessibility skills but also the ability to obtain credible health information determines individuals’ HL [19,37,39]. The first non-German replication of the Ebbinghaus forgetting experiment revealed individual differences in the lengths of memory retention intervals [40]. The Ebbinghaus forgetting curve describes forgetting over intervals ranging from 20 minutes to 31 days after information is accessed. It is a key psychology study that has affected our understanding of information literacy. According to the HL framework, some demographic factors, such as age, moderate the development of HL; however, increasing age alone could not possibly contribute to the higher HL in our study, and this result might be confounded by other factors in the multivariate analysis. In addition, participants with perceived sufficient knowledge regarding COVID-19 in this study may have higher HL, as there are reports indicating that knowledge mediates the effects of HL [41].

Regarding behaviors about COVID-19 prevention, this study found that overall HL has a weak positive correlation with drivers’ COVID-19 prevention behavior. No evidence of a correlation between judgment and application of information and COVID-19 prevention behavior was found. The finding on the positive correlation between HL and COVID-19 prevention behaviors was similar to an online survey of Vietnamese health care workers by Do et al [42], who reported a significant positive association between HL and health care workers' self-reported adherence to occupational infection prevention and control measures regarding COVID-19. In a national web-based cross-sectional survey of Chinese internet users, Li and Liu [29] also revealed that HL is significantly associated with the self-reported practice of protective behaviors against COVID-19 during the pandemic.

In a multivariate analysis to predict COVID-19 prevention behavior, we found that using Facebook to access information is a factor associated with participants’ preventive behavior level. Drivers who frequently accessed information via Facebook were more likely to exhibit excellent behavior than those who used Facebook only occasionally. Our finding was consistent with a national web-based cross-sectional survey of Chinese internet users authored by Li and Liu [29], who reported that social media use frequency significantly predicts COVID-19 prevention behaviors. It has also been reported that social media use plays a positive role in individual health behaviors [43].

Health information shared on social media has the potential to improve people’s preventive behavior toward COVID-19. However, information access skills and the ability to obtain credible health information determine individuals’ HL [19,37,39]. Library skills and internet skills, including information evaluation skills, were found to be the most essential skills related to health behaviors, and information verification is also an important skill to confirm the credibility of information before applying it in practice [44,45]. This is consistent with the finding of this study that drivers who verified information were more likely to have good COVID-19 health behaviors. It is crucial to ensure that individuals have the necessary skills to obtain and judge health information before they apply it in practice. In addition, the HL score was a factor associated with preventive behavior, and drivers with higher HL scores were more likely to have good COVID-19 prevention behaviors.

### Strengths and Limitations

The major strength of this study is that it was the first study conducted among Thai food delivery drivers in southern Thailand during the COVID-19 pandemic, exploring the drivers’ HL status and its associated factors.

This study also had some limitations. First, we used convenience sampling to recruit eligible drivers to join the study. That drivers who were interested in this study might share similar traits (eg, being in a younger population group or working in the same food delivery company) that could result in bias cannot be ruled out. Second, the results from this study may not be applicable to drivers in other regions of Thailand because of the different provincial zones categorized by the CCSA with their own specific measures to stun the spread of coronavirus. Future research conducted with a bigger sample size would likely improve the generalizability of the results. Finally, our data collection process overlapped with the COVID-19 pandemic and was performed in the late afternoon until the evening. Thus, only drivers who were available at the time participated in the study. This might have affected the external validity of this study as drivers who did not participate in the study might have had a different socioeconomic status or different HL and preventive behaviors. Future studies could use different means to distribute the survey to a more diverse audience.

### Conclusion

Most food delivery drivers reported sufficient knowledge about COVID-19. Most drivers can easily access information about handwashing and mask wearing. They were able to frequently access information and usually verified information to confirm its credibility. They had excellent HL and COVID-19 prevention behaviors. Most had good levels of understanding, judgment, and application of information regarding COVID-19. As good understanding and decision-making/judgment of information may not reflect good preventive behaviors, conventional health education alone may not be effective for COVID-19 prevention among drivers. Thus, an effective technique to apply the information to practice would enhance drivers’ HL, including COVID-19 prevention behavior. Many risk factors were observed. Information access skills alone may not determine individuals’ HL, but the ability to obtain credible health information, effective interactive communication, and knowledge could determine HL. To ensure the long-term prevention of COVID-19 in the food delivery sector, action must be taken now to eliminate the hidden barriers to health information communication with the drivers in the pandemic context. For practitioners as drivers, it is important to have sufficient support from restaurant staff, consumers, food delivery companies, and government authorities to ensure contact-free delivery and strict use of new face masks, gloves, and hand sanitizers for COVID-19 risk mitigation. Policymakers should raise awareness of safety practices for drivers through effective techniques to ensure the successful application of health information to practice, for instance, hands-on education and coaching with online learning regarding COVID-19 prevention that fits drivers' work baseline literacy. Importantly, building trust is key for successful collaboration between drivers and government authorities to enhance the drivers’ HL and preventive behaviors regarding COVID-19. A better understanding of HL among food delivery drivers would be useful for planning effective interventions for this population, especially for Thai food delivery services.

## Abbreviations

CCSA: Center for COVID-19 Situation Administration
HL: health literacy
THB: Thai baht

## References

[1] World Health Organization (2020). Novel Coronavirus – China.

[2] Zhu N, Zhang D, Wang W, Li X, Yang B, Song J, Zhao X, Huang B, Shi W, Lu R, Niu P, Zhan F, Ma X, Wang D, Xu W, Wu G, Gao GF, Tan W, China Novel Coronavirus Investigating Research Team (2020). A novel coronavirus from patients with pneumonia in China, 2019. N Engl J Med.

[3] Milne GJ, Xie S, Poklepovich D, O'Halloran D, Yap M, Whyatt D (2021). A modelling analysis of the effectiveness of second wave COVID-19 response strategies in Australia. Sci Rep.

[4] Dsouza D, Sharma D (2020). Online food delivery portals during COVID-19 times: an analysis of changing consumer behavior and expectations. IJIS.

[5] Li C, Mirosa M, Bremer P (2020). Review of online food delivery platforms and their impacts on sustainability. Sustainability.

[6] Zanetta LD, Hakim MP, Gastaldi GB, Seabra LMJ, Rolim PM, Nascimento LGP, Medeiros CO, da Cunha DT (2021). The use of food delivery apps during the COVID-19 pandemic in Brazil: the role of solidarity, perceived risk, and regional aspects. Food Res Int.

[7] Chang H, Meyerhoefer C (2020). COVID-19 and the demand for online food shopping services: empirical evidence from Taiwan. Am J Agric Econ.

[8] Byrd K, Her E, Fan A, Almanza B, Liu Y, Leitch S (2021). Restaurants and COVID-19: what are consumers' risk perceptions about restaurant food and its packaging during the pandemic?. Int J Hosp Manag.

[9] Ortiz-Prado E, Henriquez-Trujillo AR, Rivera-Olivero IA, Lozada T, Garcia-Bereguiain MA, UDLA-COVID-19 team (2021). High prevalence of SARS-CoV-2 infection among food delivery riders. A case study from Quito, Ecuador. Sci Total Environ.

[10] Felicia (2021). Thailand's Best Food Delivery Service.

[11] Sirikeratikul S (2020). Thailand Online Food Delivery Market.

[12] Burdorf A, Porru F, Rugulies R (2020). The COVID-19 (coronavirus) pandemic: consequences for occupational health. Scand J Work Environ Health.

[13] Nguyen THD, Vu DC (2020). Food delivery service during social distancing: proactively preventing or potentially spreading coronavirus disease-2019?. Disaster Med Public Health Prep.

[14] Liu C, Cao S Beijing Deliveryman Who Sends 50 Orders per Day Confirmed with COVID-19.

[15] Nguyen THD, Vu DC (2020). The largest epicenter of the coronavirus outbreak in Vietnam. Infect Control Hosp Epidemiol.

[16] Hindustan Times (2020). Food Delivery Agent Tests Covid-19 +ve in Delhi, 72 Families Quarantined.

[17] Centers for Disease Control and Prevention (2020). What Food and Grocery Pick-Up and Delivery Drivers Need to Know about COVID-19.

[18] Baker DW (2006). The meaning and the measure of health literacy. J Gen Intern Med.

[19] Sørensen K, Van den Broucke S, Fullam J, Doyle G, Pelikan J, Slonska Z, Brand H, (HLS-EU) Consortium Health Literacy Project European (2012). Health literacy and public health: a systematic review and integration of definitions and models. BMC Public Health.

[20] Liu H, Zeng H, Shen Y, Zhang F, Sharma M, Lai W, Zhao Y, Tao G, Yuan J, Zhao Y (2018). Assessment tools for health literacy among the general population: a systematic review. Int J Environ Res Public Health.

[21] Klamklay J, Sungkhapong A, Yodpijit NE, E. Patterson P (2008). Anthropometry of the southern Thai population. Int J Ind Ergon.

[22] Situation Awareness Team (SAT), Department of Disease Control (2022). COVID-19 Situation in Southern Thailand.

[23] Harvard Humanitarian Initiative (2021). KoBo Toolbox.

[24] Pourhoseingholi MA, Vahedi M, Rahimzadeh M (2013). Sample size calculation in medical studies. Gastroenterol Hepatol Bed Bench.

[25] Marques S, Lemos S (2017). Health literacy assessment instruments: literature review. Audiol Commun Res.

[26] Khasawneh AI, Humeidan AA, Alsulaiman JW, Bloukh S, Ramadan M, Al-Shatanawi TN, Awad HH, Hijazi WY, Al-Kammash KR, Obeidat N, Saleh T, Kheirallah KA (2020). Medical students and covid-19: knowledge, attitudes, and precautionary measures. a descriptive study from Jordan. Front Public Health.

[27] Olaimat AN, Aolymat I, Shahbaz HM, Holley RA (2020). Knowledge and information sources about covid-19 among university students in Jordan: a cross-sectional study. Front Public Health.

[28] Hamza MS, Badary OA, Elmazar MM (2021). Cross-sectional study on awareness and knowledge of covid-19 among senior pharmacy students. J Community Health.

[29] Li X, Liu Q (2020). Social media use, eHealth literacy, disease knowledge, and preventive behaviors in the covid-19 pandemic: cross-sectional study on Chinese netizens. J Med Internet Res.

[30] Yang K, Liu H, Ma L, Wang S, Tian Y, Zhang F, Li Z, Song Y, Jiang X (2021). Knowledge, attitude and practice of residents in the prevention and control of COVID-19: an online questionnaire survey. J Adv Nurs.

[31] Ulla M, Perales W (2021). Facebook as an integrated online learning support application during the COVID19 pandemic: Thai university students' experiences and perspectives. Heliyon.

[32] Archila PA, Danies G, Molina J, Truscott de Mejía A-M, Restrepo S (2021). Towards covid-19 literacy: investigating the literacy levels of university students in Colombia. Sci Educ (Dordr).

[33] González-Padilla DA, Tortolero-Blanco L (2020). Social media influence in the covid-19 pandemic. Int Braz J Urol.

[34] Mackenbach JP (2012). The persistence of health inequalities in modern welfare states: the explanation of a paradox. Soc Sci Med.

[35] Okan O, Bollweg TM, Berens E, Hurrelmann K, Bauer U, Schaeffer D (2020). Coronavirus-related health literacy: a cross-sectional study in adults during the COVID-19 infodemic in Germany. Int J Environ Res Public Health.

[36] Ministry of Foreign Affairs, Kingdom of Thailand (2021). Regulation Issued under Section 9 of the Emergency Decree on Public Administration in Emergency Situations B.E. 2548 (2005) (No. 22).

[37] Suka M, Odajima T, Okamoto M, Sumitani M, Igarashi A, Ishikawa H, Kusama M, Yamamoto M, Nakayama T, Sugimori H (2015). Relationship between health literacy, health information access, health behavior, and health status in Japanese people. Patient Educ Couns.

[38] Pechrapa K, Yodmai K, Kittipichai W, Charupoonpol P, Suksatan W (2021). Health literacy among older adults during covid-19 pandemic: a cross-sectional study in an urban community in Thailand. Ann Geriatr Med Res.

[39] Nutbeam D (2008). The evolving concept of health literacy. Soc Sci Med.

[40] Murre JMJ, Dros J (2015). Replication and analysis of Ebbinghaus' forgetting curve. PLoS One.

[41] Lee S, Arozullah A, Cho Y (2004). Health literacy, social support, and health: a research agenda. Soc Sci Med.

[42] Do BN, Tran TV, Phan DT, Nguyen HC, Nguyen TTP, Nguyen HC, Ha TH, Dao HK, Trinh MV, Do TV, Nguyen HQ, Vo TT, Nguyen NPT, Tran CQ, Tran KV, Duong TT, Pham HX, Nguyen LV, Nguyen KT, Chang PWS, Duong TV (2020). Health literacy, eHealth literacy, adherence to infection prevention and control procedures, lifestyle changes, and suspected covid-19 symptoms among health care workers during lockdown: online survey. J Med Internet Res.

[43] Laranjo L, Arguel A, Neves AL, Gallagher AM, Kaplan R, Mortimer N, Mendes GA, Lau AYS (2015). The influence of social networking sites on health behavior change: a systematic review and meta-analysis. J Am Med Inform Assoc.

[44] Ivanitskaya LV, Hanisko KA, Garrison JA, Janson SJ, Vibbert D (2012). Developing health information literacy: a needs analysis from the perspective of preprofessional health students. J Med Libr Assoc.

[45] Shehata A (2020). Health information behaviour during COVID-19 outbreak among Egyptian library and information science undergraduate students. Inf Dev.

